# Adenine Methylation in *Drosophila* Is Associated with the Tissue-Specific Expression of Developmental and Regulatory Genes

**DOI:** 10.1534/g3.119.400023

**Published:** 2019-04-15

**Authors:** Kinnary Shah, Weihuan Cao, Christopher E. Ellison

**Affiliations:** Department of Genetics, Human Genetics Institute of New Jersey, Rutgers, The State University of New Jersey, Piscataway, New Jersey

**Keywords:** *Drosophila* methylation epigenetics 6mA nanopore

## Abstract

N6-methyladenine (6mA or m6dA) is a DNA modification that has long been known to play an important role in a variety of biological functions in prokaryotes. This modification has only recently been described in eukaryotes, where it seems to have evolved species-specific functions ranging from nucleosome positioning to transposon repression. In *Drosophila*, 6mA has been shown to be important for enforcing the tissue specificity of neuronal genes in the brain and suppressing transposable element expression in the ovaries. In this study, we have analyzed the raw signal data from nanopore sequencing to identify 6mA positions in the *D. melanogaster* genome at single-base resolution. We find that this modification is enriched upstream from transcription start sites, within the introns and 3′ UTRs of genes, as well as in simple repeats. These 6mA positions are enriched for sequence motifs that are recognized by known transcriptional activators involved in development, such as Bicoid and Caudal, and the genes that carry this modification are enriched for functions involved in development, regulation of transcription, and neuronal activity. These genes show high expression specificity in a variety of tissues besides the brain, suggesting that this modification may play a more general role in enforcing the specificity of gene expression across many tissues, throughout development, and between the sexes.

The DNA modification N6-methyladenine (6mA) is common among prokaryotes and is known to play a role in the restriction/modification systems involved in defense against bacteriophage infection (Luria and Human 1952), as well as in the regulation of gene expression (Oshima *et al.* 2002; Reisenauer and Shapiro 2002). This modification has only recently been described in eukaryotes, where it has been implicated in a variety of functions. In the ciliate *Tetrahymena thermophila* and the green alga *Chlamydomonas*, 6mA is associated with active transcription and may play a role in nucleosome positioning (Fu *et al.* 2015; Wang *et al.* 2017; Luo *et al.* 2018). In humans, the modification is also associated with active transcription and is enriched in exons (Xiao *et al.* 2018). In *C. elegans*, 6mA is broadly distributed across the genome and its exact function remains unclear (Greer *et al.* 2015), whereas in *Drosophila* and mice, there is evidence that the 6mA modification is involved in suppression of transposable elements and transcriptional repression in neurons (Zhang *et al.* 2015; Yao *et al.* 2017; Yao *et al.* 2018). 6mA is most prevalent in the early *Drosophila* embryo, where it may play a role in development, but this modification has also been identified in ovary and brain tissues (Zhang *et al.* 2015; Yao *et al.* 2018). In *Drosophila* ovaries, there is evidence that 6mA is involved in transposon suppression, while in the brain, 6mA acts in concert with polycomb group proteins to repress gene expression (Zhang *et al.* 2015; Yao *et al.* 2018). A 6mA methyltransferase has not been identified in *Drosophila*, however the ten-eleven-translocation (TET) protein, DMAD, acts as a demethylase that facilitates the removal of 6mA (Zhang *et al.* 2015). Recent work has shown that DMAD null mutants accumulate 6mA methylation in the brain and have defects in brain development, suggesting that removal of the 6mA modification by DMAD is required for gene activation and proper brain development (Yao *et al.* 2018). Based on these results, Yao *et al.* (2018) describe a model for 6mA function where this modification enforces transcriptional silencing of a set of neuronal genes outside of the brain. Within neurons, DMAD acts in combination with the transcriptional activator Wds to remove 6mA from these genes, leading to their neuron-specific expression.

Based on these results, we hypothesized that the 6mA modification might play a more general role in enforcing the tissue-specific expression of a much larger set of genes across multiple tissues in the adult fly. To test this hypothesis, we analyzed the raw signal data generated by nanopore sequencing of *Drosophila melanogaster* adult females, to identify a set of high-confidence genomic positions where the 6mA modification is present in the majority of cells in the adult fly. We find that these positions are enriched at simple repeats and within the introns, 3′ UTRs, and upstream regions of genes. The genes that carry these modifications are highly enriched for developmental, regulatory, and neuronal functions, they are expressed in a variety of tissues, and their expression is significantly more tissue specific than unmethylated genes.

## Materials & Methods

### Nanopore Sequencing

DNA was extracted from 30 adult females using the Qiagen DNeasy Blood & Tissue Kit and prepped for sequencing using the Oxford Nanopore Technologies (ONT) SQK-LSK108 library preparation kit. The PCR-free libraries were constructed using the ONT *1D Genomic DNA by Ligation* protocol and the PCR-based library was constructed using the ONT *1D Low Input Genomic DNA with PCR* protocol. Libraries were sequenced on the MinION Mk1B device using version r9.4 flow cells and basecalled using the ONT *Albacore* software package (version 2.1.10).

### Genome Assembly

We used *Canu* (Koren *et al.* 2017) to assemble the DGRP732 nanopore reads using an estimated genome size of 140 Mb along with the options: *overlapper = mhap utgReAlign = true*. We generated Hi-C data from ∼200 mg of 8-16 hr embryos using a *in situ* DNase Hi-C protocol (Ramani *et al.* 2016), aligned the data to our Canu assembly using *Juicer* (Durand *et al.* 2016), and scaffolded the Canu contigs using the *3D-DNA* pipeline (Dudchenko *et al.* 2017). We used *3D-DNA* to create a single “megascaffold”, which we then manually separated into chromosome arms based on comparisons to the *D. melanogaster* reference assembly. We polished our scaffolds using nanopore reads with *Racon* (Vaser *et al.* 2017) and then identified uninformative (*i.e.*, reads that did not contain a ligation junction) Illumina reads from our Hi-C data, which we used as single-end reads to polish the assembly with *Pilon* (Walker *et al.* 2014). We used *Mercator* (Dewey 2007) to create a one-to-one orthology map between assemblies.

### Wolbachia

We used *BLAST* (Altschul *et al.* 1990) to search the *wMel Wolbachia* genome assembly against our DGRP732 assembly to determine if any assembled contigs were from *Wolbachia*. We also searched for *Wolbachia*-derived nanopore sequences by aligning the raw reads to the *wMel* genome assembly using *graphmap* (Sović *et al.* 2016). We observed high rates of cross-mapping of *Drosophila* reads to two different ∼100kb segments of the *wMel* assembly when mapping all reads to the *wMel* assembly alone. We therefore created a concatenated genome assembly composed of both the DGRP732 and *wMel* assemblies. We used *bedtools* to mask the two ∼100kb segments of the *wMel* genome and *samtools* (Li *et al.* 2009) to exclude alignments with mapping quality < 20. We then used *bedtools* to calculate the average read coverage for the remainder of the *Wolbachia* genome.

### 6mA Identification

We used the genome assembly described above and aligned the raw signal data to the assembly using the re-squiggle algorithm in *Tombo* (version 1.4)(Stoiber *et al.* 2017). We then calculated sequencing coverage for each genomic position, for each dataset, and removed positions whose coverage fell outside of +/− 50% of the genomic mean. We used the Tombo 6mA model (command: *tombo detect_modifications alternative_model–alternate-bases 6mA*) to identify A/T positions whose signal level matched a 6mA model better than the canonical base. We did this for each of the two PCR-free sequencing replicates and retained positions where at least 70% of reads were inferred to carry the 6mA modification in replicate 1 and whose percentage estimate was replicated in the second sequencing sample (within +/− 10%). We also ran *Tombo* with a control dataset using the *sample_compare* algorithm to identify all positions whose signal deviated from the expected level (determined by the control library)(command: *tombo detect_modifications sample_compare*) and retained positions with at least 70% modified reads. We used *bedtools* (Quinlan and Hall 2010) to identify the positions that were retained in both the 6mA model and *sample_compare* approaches, which became our set of 10,467 high confidence 6mA positions. We followed an analogous approach to also identify 1,648,942 A/T positions where we were confident that they did NOT contain the 6mA modification, which we used as control positions for our genome features permutation test.

### Genome Features Enrichment

We transferred the coordinates of our high-confidence positions from the DGRP732 assembly to the *D. melanogaster* reference genome assembly FlyBase version 6 (Hoskins *et al.* 2015; Thurmond *et al.* 2019) by using *Mercator* (Dewey 2007) to create a whole-genome alignment between the two assemblies. We used the longest isoform per gene from the FlyBase r6.22 genome feature annotations along with *bedtools* to count the number of positions that overlapped each of the features shown in Figure 2A. TE insertion and simple repeat locations will differ between our strain and the iso1 reference assembly. To determine whether these differences affect our enrichment analysis, we identified TE insertions and simple repeats in both the iso1 and DGRP732 assemblies using *RepeatMasker* (www.repeatmasker.org) and *tantan* (Frith 2011) and determined the number of 6mA positions that overlap these features in each assembly. We found the same enrichment patterns in both cases. To determine the statistical significance of enriched or depleted features, we randomly sampled a set of 10,467 positions from our control positions (*i.e.*, those that do not contain the 6mA modification) without replacement and counted the number of features for each annotation category that they overlapped. We repeated this resampling procedure 10,000 times and calculated the p-value using the number of times the counts from the random set were greater than or equal to the counts from our high-confidence positions.

### Motif Enrichment

We used *bedtools* to extract +/− 25 bp of sequence surrounding each high confidence position and the findMotifs.pl script from *Homer* (Heinz *et al.* 2010) to identify enriched motifs in these sequences. For the background set of sequences, we used +/− 25 bp surrounding the set of control positions which we determined did not contain the 6mA modification. Similarity to known sequence motifs was reported by findMotifs.pl. To verify the *Homer* motifs, we used the differential enrichment function from *MEME* (Bailey *et al.* 2009) on the same set of sequences that we used for *Homer* (Heinz *et al.* 2010). For the *MEME* search, we used an E-value threshold of 0.1 and min/max motif size of 8 and 12, respectively. We used *STAMP* (Mahony and Benos 2007) to compare the enriched motifs identified by *Homer* to those identified by *MEME*.

### 6mA-containing Genes

We used *bedtools* along with the FlyBase gene annotations to identify every gene that overlapped one or more 6mA positions. We identified enriched GO terms for this gene set using GOrilla (Eden *et al.* 2009) with all FlyBase genes as the background set. We obtained expression enrichment scores for these genes from FlyAtlas (Chintapalli *et al.* 2007).

### Data availability

All sequencing data and the DGRP732 genome assembly are available via the NCBI Sequence Read Archive (SRA) and Whole Genome Shotgun (WGS) databases under Bioproject PRJNA515844. Supplemental material available at FigShare: https://doi.org/10.25387/g3.7841036.

## Results

### Genome Assembly

We performed nanopore sequencing of DNA extracted from 30 adult females of strain DGRP-732 from the *Drosophila* Genetic Reference Panel (DGRP)(Mackay *et al.* 2012)(Table S1). We assembled the nanopore data using *Canu* (Koren *et al.* 2017), scaffolded the *Canu* contigs with Hi-C data using the *3D-DNA* pipeline (Dudchenko *et al.* 2017), and polished the scaffolds using *Racon* (Vaser *et al.* 2017) and *Pilon* (Walker *et al.* 2014). We obtained contig/scaffold N50 values of 5.4 Mb and 25.7 Mb, respectively and identified one-to-one alignments between our assembly and the *D. melanogaster* iso1 reference genome (Hoskins *et al.* 2015) that encompassed 98% of the iso1 assembly. The assembly has been deposited in the NCBI whole genome shotgun (WGS) database (BioProject PRJNA515844) and will be described in more detail in another manuscript (Ellison & Cao, in prep).

### Identification and genomic locations of 6mA modifications

**Figure 1:**
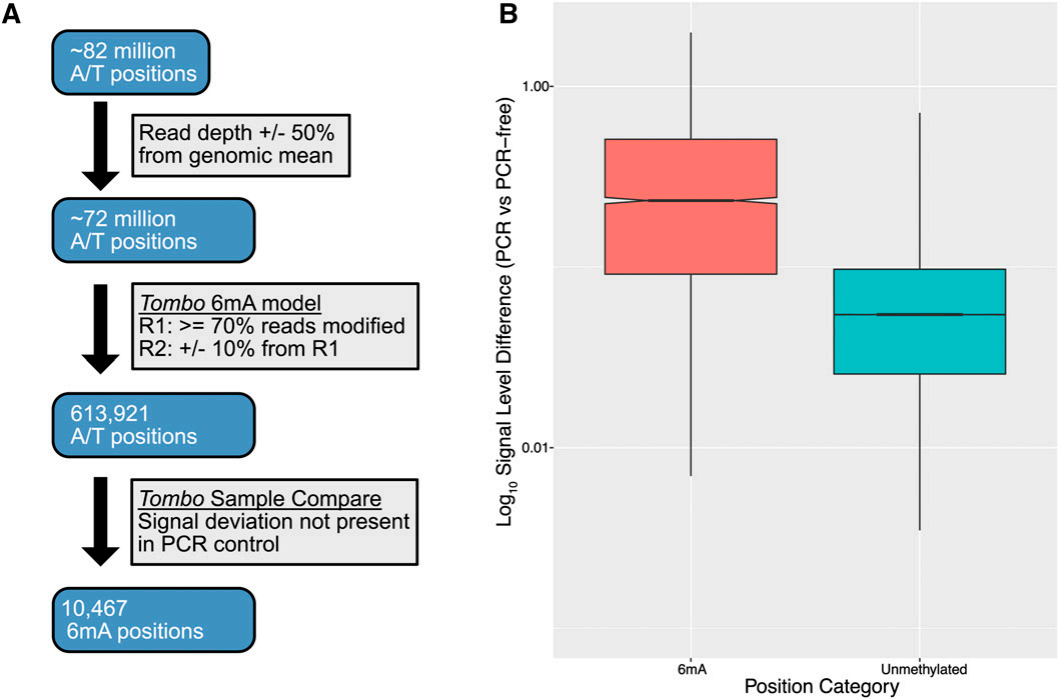
Identification of 6mA positions in the *Drosophila melanogaster* genome. Panel A: Flowchart showing the filtering steps involved in identifying the high-confidence 6mA positions. Panel B: Boxplot showing the difference in signal level between the PCR-free and control sequencing libraries for the high-confidence 6mA positions and unmethylated positions. As expected, the adenine positions that were identified as methylated show a significantly larger difference in signal level compared to the unmethylated positions (Wilcoxon Test *P* < 2.2e-16).

We performed nanopore sequencing in two replicates (one flowcell per replicate) and used the 6mA model in the *Tombo* software package (Stoiber *et al.* 2017) to estimate the percent of sequences carrying the 6mA modification for each genomic position (percent reads modified, PRM). We found only a moderately strong correlation in PRM values between replicates (Spearman rho = 0.56), which suggests that there is a fair amount of noise in the raw nanopore signal data. For this reason, we used a series of stringent criteria for identifying putative 6mA positions. We started by only considering positions with a PRM value >= 70% in replicate 1 whose PRM value was conserved in our second sequencing experiment (+/− 10%), which resulted in 613,921 genomic positions (Figure 1A).

These two sequencing libraries were generated using a PCR-free protocol in order to preserve the 6mA modifications. We also generated a third library as a control using a PCR-based protocol in order to produce sequences from DNA molecules lacking the 6mA modification. We sequenced this library using a single flowcell and then used the *sample compare* model in *Tombo* to identify all genomic positions whose PCR-free current levels deviated from those in the control library. We intersected these positions with those identified using the 6mA model, to create a final set of 10,467 high-confidence 6mA positions (Figure 1A, File S1). We also used an analogous approach to identify a set of positions where we had high-confidence that they did *not* carry the 6mA modification (6mA-free positions). As expected, we find a much larger difference in current level between the PCR and PCR-free libraries for the 6mA positions compared to the 6mA-free positions (Wilcoxon-test *P* < 2.2e-16, Figure 1B). To determine whether 6mA positions are conserved between *Drosophila* strains, we performed nanopore sequencing of a PCR-free library from strain DGRP379 using a single flowcell and identified all genomic positions with a PRM value >= 70%. We found that, for ∼16% of our high-confidence positions from DGRP732, the exact same position is identified as putatively methylated in DGRP379, which is significantly more than expected by chance (hypergeometric *P* < 2.2e-16). Overall, ∼90% of the high-confidence positions from DGRP732 are within 100bp of a putatively methylated position in DGRP379, suggesting that, between strains, the methylation status of a given genomic region is more conserved than that of specific basepairs.

Both DGRP379 and DGRP732 were previously reported to lack the endosymbiotic bacterium *Wolbachia* that is common among *Drosophila* (Richardson *et al.* 2012). To verify that our stocks were also uninfected, we first confirmed via BLAST that the DGRP732 assembly lacked contigs showing homology to *Wolbachia*. We then appended the *wMel Wolbachia* genome assembly (Wu *et al.* 2004) to the DGRP732 assembly, aligned the raw nanopore reads from each strain to the concatenated assembly, and calculated the average coverage for the *Wolbachia* genome using only reads with mapping quality >= 20 (see Methods). For DGRP379, we found only three reads that mapped to *Wolbachia* (average coverage = 0.004x) and for DGRP739, which has more sequencing data, we found 44 reads (average coverage = 0.19x). Based on these results, we conclude that *Wolbachia* is either absent from these strains, as previously reported, or at very low abundance.

**Figure 2:**
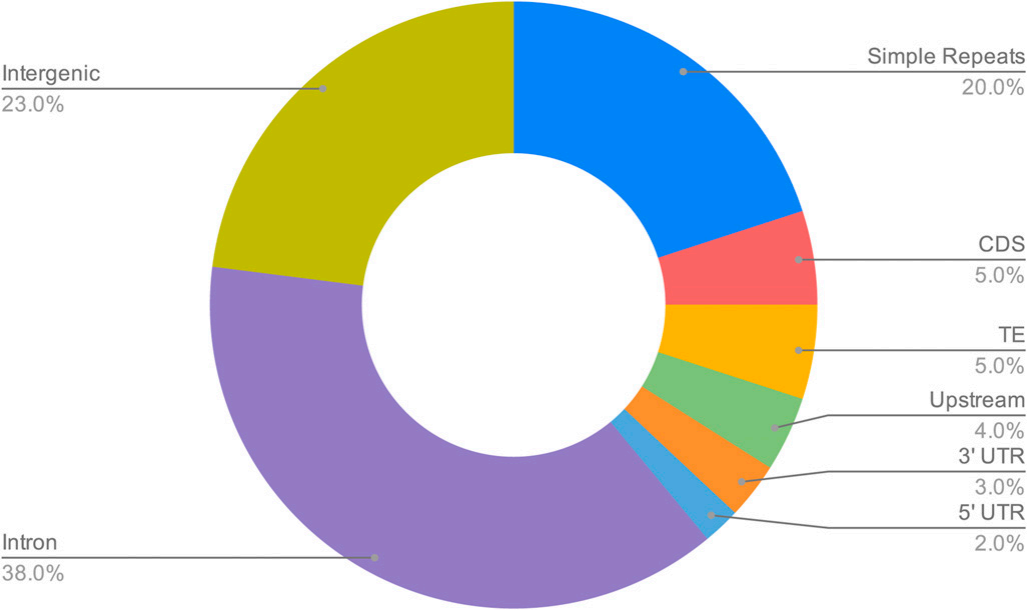
Genomic features overlapping 6mA positions. The doughnut chart shows the proportion of 6mA positions within each genome feature category. From a permutation test (see Methods), we found that the 6mA positions overlap introns, 3′ UTRs, upstream from transcription start sites, and simple repeats significantly more than expected by chance, whereas they are significantly depleted from transposable elements as well as coding and intergenic sequences (3′ UTR: *P* = 0.0079, *P* < 0.0001 in all other cases).

We investigated the genomic enrichment of our DGRP732 high-confidence 6mA-methylated sites by calculating the number of positions that overlap the following features: CDS, introns, 5′ UTR, 3′ UTR, 1kb upstream, intergenic sequence, simple repeats, and transposable elements. Using a permutation test (see Methods), we found that our 6mA positions are significantly enriched at introns, 3′ UTRs, upstream from transcription start sites, and simple repeats, whereas they are significantly depleted from coding sequence, intergenic sequence, and transposable elements (TEs)(3′ UTR: *P* = 0.0079, *P* < 0.0001 in all other cases)(Figure 2). A similar enrichment pattern was previously reported for gain-of-6mA positions in the brain of a DMAD mutant (Yao *et al.* 2018), with the exception of TEs, which we find as being depleted of 6mA whereas they are weakly enriched within TEs in the brain (Yao *et al.* 2018). In another study, 6mA positions were highly enriched within TEs in the ovaries (Zhang *et al.* 2015). We also found that, among chromosomes, the 6mA positions showed less than a twofold difference in their density, varying from ∼5 positions per 100kb (chromosomes 4 & 3R) to ∼10 positions per 100kb (chromosome 2L), on average (Figure S1A). Within chromosomes, the 6mA positions were fairly evenly distributed across the euchromatic chromosome arms and depleted from the pericentric heterochromatin (Figure S1B).

Previous work identified gain-of-6mA genomic regions in the brain that showed an accumulation of 6mA methylation in a DMAD null mutant. We found 3% (318 positions) of our high-confidence positions overlap these gain-of-6mA locations, which is significantly more than expected by chance (hypergeometric test *P* = 3.2e-13).

Our final set of 6mA positions show an enrichment pattern similar to what was previously observed in the brain (Yao *et al.* 2018). They also show a signficant overlap with the gain-of-6mA positions identified in a DMAD mutant (Yao *et al.* 2018) and their locations are conserved between DGRP strains. These results suggest that, despite the noise in the raw nanopore signal data, our conservative approach of using replicates and a control dataset has allowed us to accurately identify 6mA positions at single base resolution.

### 6mA sequence motifs

**Figure 3:**
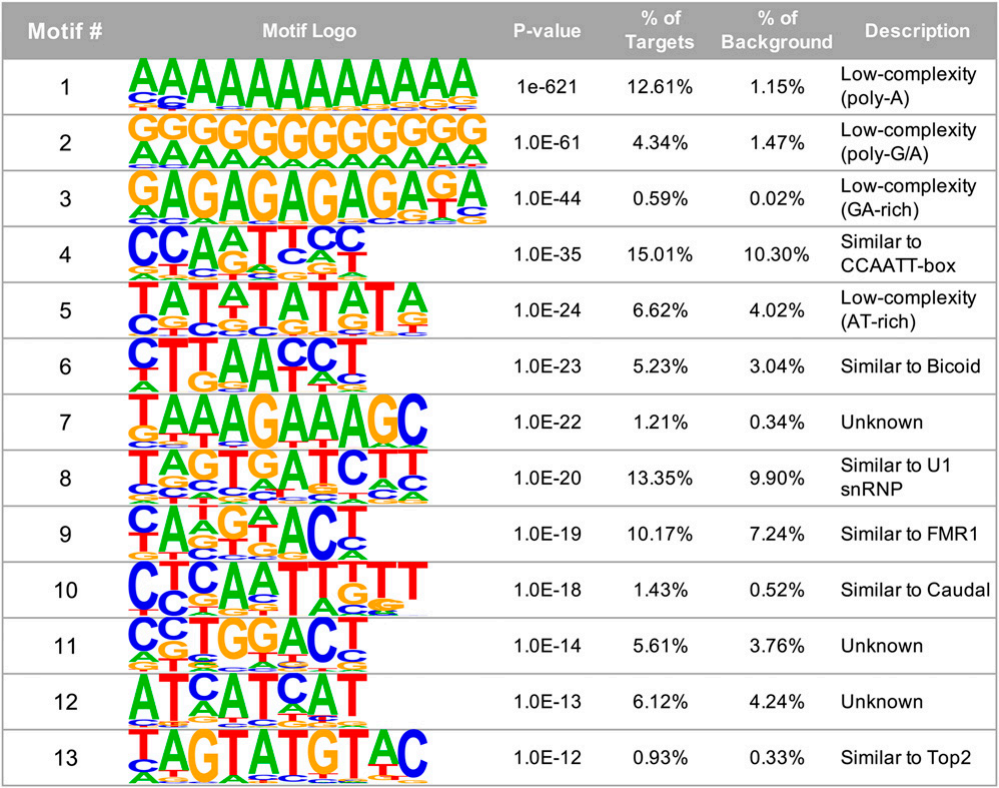
Sequence motifs associated with 6mA positions. We extracted 50 bp of sequence surrounding each 6mA position and used *Homer* (Heinz *et al.* 2010) to search for enriched motifs within these sequences. Four of the motifs identified by *Homer* are low-complexity sequences, including two GA-rich sequences (motifs 1, 2, 3, and 5). Three motifs are similar to those recognized by developmental transcription factors including the CBF family, Bicoid, and Caudal (motifs 4, 6, and 10), and three others are similar to motifs recognized by genes involved in RNA processing: U1 snRNP, Fmr1, Top2 (motifs 8, 9, and 13).

We extracted a total of 50 basepairs surrounding each 6mA position and used *Homer* (Heinz *et al.* 2010) to identify enriched motifs present in the sequences. We identified a total of 13 significantly enriched motifs (Figure 3). Four of these motifs consist of low-complexity sequences, including a poly-A motif, two GA-rich motifs, and a TA-repeat (Figure 3: motifs 1, 2, 3, and 5). Interestingly, the 6mA-associated sequence motifs previously identified in *C. elegans* (AGAAGAAGAAGA)(Greer *et al.* 2015) and the *Drosophila* brain (AGAAGGAG)(Yao *et al.* 2018) are also GA-rich elements. Two of the motifs that we identify (motifs 6 and 10) are very similar to the motifs that are recognized by Bicoid (CTAATCT) and Caudal (AAATTTTT), which are both homeobox transcription factors involved in anterior/posterior patterning (Mlodzik and Gehring 1987; Driever and Nusslein-Volhard 1989). We also find a motif (motif 8) that resembles the sequence recognized by the small ribonucleoprotein particle U1 (subunit 70K)(TCTTGATC)(Mancebo *et al.* 1990), which is part of the spliceosome, and another motif (motif 4) that contains the sequence CCAAT, which is commonly found in eukaryotic promoters and is recognized by CBF domain transcription factors (Bucher 1990). In *Drosophila*, the three CBF transcription factors (Nf-YA, Nf-YB, and Nf-YC) have been shown to play a role in eye and thorax development (Yoshioka *et al.* 2008; Ly *et al.* 2013). Another of our enriched motifs (motif 9) is similar to that recognized by Fmr1 (ANGGACA), which is involved in RNA trafficking and translation, and whose loss causes Fragile X syndrome (Ishizuka *et al.* 2002), while another motif (motif 13) is similar to that recognized by Topoisomerase 2 (Top2; TACATATGTATGTA), which is well-known for its role in DNA replication, but has also been found to play a role in transcription and insulator function (Lupo *et al.* 2001; Ramos *et al.* 2011). To verify these motifs, we also ran *MEME* (Bailey *et al.* 2009) using the same input sequences. We compared the *Homer* *vs.* *MEME* enriched motifs using *STAMP* (Mahony and Benos 2007) and found that 12 of the 13 *Homer* motifs show significant similarity to one or more motifs identified by *MEME* (Table S2). The only motif that was not identified by *MEME* is *Homer* motif #8 (Figure 3). Together, these motifs suggest that the 6mA modification plays a role in regulating development and transcription and raise the possibility that DMAD may interact with other transcriptional activators besides Wds.

### 6mA is enriched at developmental genes

Our set of 6mA positions overlap a total of 2,624 genes (File S1). We performed a Gene Ontology (GO) enrichment analysis on this set of genes and found a total of 549 significantly enriched terms with FDR q-value <= 0.02 across the three GO categories (COMPONENT: 57, FUNCTION: 71, PROCESS: 421)(Table S3). The enriched terms are heavily biased toward categories involved in development, regulation of gene expression, and neuron-specific functions (Table 1), in agreement with the previously identified roles for this modification in *Drosophila* (Zhang *et al.* 2015; Yao *et al.* 2018).

**Table 1:** GO term enrichment for genes containing the 6mA modification

GO term ID*	Description	FDR q-value
**PROCESS**		
GO:0032502	developmental process	5.49E-36
GO:0007411	axon guidance	1.25E-22
GO:0050793	regulation of developmental process	2.35E-20
GO:0009886	post-embryonic animal morphogenesis	3.63E-19
GO:0010646	regulation of cell communication	5.03E-16
GO:0023051	regulation of signaling	4.82E-16
GO:0022414	reproductive process	1.39E-07
GO:0010468	regulation of gene expression	2.17E-06
**FUNCTION**		
GO:0005261	cation channel activity	3.07E-06
GO:0003700	DNA-binding transcription factor activity	5.50E-06
GO:0004672	protein kinase activity	1.63E-04
GO:0003779	actin binding	1.54E-04
GO:0140110	transcription regulator activity	2.40E-04
GO:0038023	signaling receptor activity	8.22E-04
**COMPONENT**		
GO:0005886	plasma membrane	9.23E-21
GO:0030054	cell junction	7.72E-12
GO:0097458	neuron part	5.91E-09
GO:1902495	transmembrane transporter complex	1.40E-08
GO:0045202	synapse	1.40E-06

* Selected GO terms are shown here. See Table S3 for full list of all 549 enriched terms.

### 6mA-containing genes show high tissue-specificity in a variety of tissues

**Figure 4:**
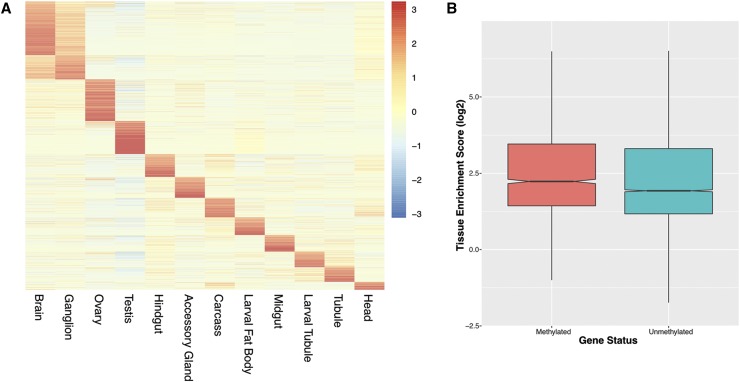
6mA positions are found in genes with high tissue specificity. The FlyAtlas project calculated enrichment scores (tissue *vs.* whole fly gene expression) for twelve different *D. melanogaster* tissues (Chintapalli *et al.* 2007). 2,051 of the 2,624 genes that overlap 6mA positions had enrichment scores, which are shown in the heatmap in Panel A, where each row represents a 6mA-containing gene and each column corresponds to one of the 12 tissues. Individual cells are colored according to enrichment score. Genes with the 6mA modification show significantly higher tissue enrichment scores compared to the *D. melanogaster* genes that do not contain any 6mA positions (Panel B; Wilcoxon Test *P* < 2.2e-16).

We used the FlyAtlas resource (Chintapalli *et al.* 2007) to examine the expression patterns of the 2,624 genes that overlap one or more 6mA positions. The FlyAtlas project used microarrays to measure gene expression in a variety of *D. melanogaster* tissues as well as in the whole fly. Part of the project involved the calculation of enrichment scores that measure the tissue specificity of each gene by comparing its expression level in a given tissue to its expression level in the whole fly (Chintapalli *et al.* 2007). Enrichment scores for 2,051 of the 2,624 genes were available from FlyAtlas and visualization of these scores shows that most genes are strongly expressed in a single tissue, with the exception of the brain and ganglia whose profiles overlap because these tissues are both part of the central nervous system (Figure 4A). Overall, we find that the 6mA-containing genes show significantly higher tissue specificity compared to the rest of the genes in the *D. melanogaster* genome (Wilcoxon test *P* < 2.2e-16)(Figure 4B). While many 6mA-containing genes are highly expressed in either the brain/ganglion or ovaries, both of which are tissues where 6mA-mediated gene regulation has previously been identified, we also find many genes whose expression is highly enriched in other tissues from both adult flies and larvae, including the testes, which suggests that 6mA may play a more general role in regulating the specificity of gene expression across many tissues and developmental stages, as well as between sexes.

## Discussion

We have used nanopore sequencing to identify 6mA methylation at single base-pair resolution across the *Drosophila* genome. These positions tend to be located upstream from transcription start sites and within introns, 3′ UTRs, and simple repeats and they are associated with genes whose functions are related to transcriptional regulation, development, and neuronal activity. These findings corroborate those from previous studies where ChIP-seq was used to identify 6mA in *Drosophila* brains and ovaries (Zhang *et al.* 2015; Yao *et al.* 2018), and provide additional support for the importance of this modification in the repression of developmental and neuronal genes. One difference between this study and previous studies of 6mA in *Drosophila* is that our 6mA positions were significantly depleted from transposable elements whereas 6mA positions in ovaries were enriched within TEs (Zhang *et al.* 2015), as were gain-of-6mA positions in the brain that became methylated in the DMAD mutant (Yao *et al.* 2018). This difference could be due to the fact that TE repression is reduced in somatic cells compared to the germline. For example, the gain-of-6mA at TEs in the brain of DMAD mutants suggests that, in wild-type flies, DMAD plays a role in actively removing 6mA from TEs in the brain (Yao *et al.* 2018). Other studies have shown that, in the embryo, TE insertions are strongly enriched for repressive histone modifications, but this enrichment weakens throughout development into adulthood (Lee 2015; Lee and Karpen 2017). Because our data are from whole, adult flies, we may not be able to identify the 6mA positions whose presence within TEs is restricted to the germline.

Our results also expand the role of 6mA in several important ways. A previous study in the *Drosophila* brain identified a single GA-rich sequence motif associated with 6mA and showed that the 6mA demethylase DMAD interacts with the regulatory protein Wds to remove 6mA and activate gene expression (Yao *et al.* 2018). While we find a similar GA-rich motif, we also find a variety of additional enriched motifs, several of which are similar to motifs recognized by regulatory proteins involved in development (Bicoid, Caudal, and CBF transcription factors) and RNA processing (U1 snRP, Fmr1, and Top2). These results suggest that 6mA may be found in different sequence contexts in different tissues and raise the possibility that DMAD may work in concert with other regulatory proteins besides Wds.

Our results also suggest that, rather than only regulating the expression of neuronal genes, the 6mA modification may play a more general role in enforcing the tissue specificity of gene expression across many different tissues, and throughout development. Given that our sequencing data were from DNA extracted from whole flies, the 6mA positions that we identify are those that carry the 6mA modification in the majority of cells in the organism. The fact that our 6mA-containing genes show high expression specificity is consistent with a model where 6mA is involved in the repression of these genes in the majority of cells. Such genes would only be expressed in a minority of cells (*e.g.*, in specific tissues, sexes, and/or developmental stages) when DMAD, in concert with one or more activation proteins, removes the modification and activates the gene. Future work involving nanopore sequencing of different *Drosophila* tissues would provide insight into this model.
